# The impact of depression and anxiety treatment on biological aging and metabolic stress: study protocol of the MOod treatment with antidepressants or running (MOTAR) study

**DOI:** 10.1186/s12888-019-2404-0

**Published:** 2019-12-30

**Authors:** Bianca A. Lever-van Milligen, Josine E. Verhoeven, Lianne Schmaal, Laura S. van Velzen, Dóra Révész, Catherine N. Black, Laura K. M. Han, Melany Horsfall, Neeltje M. Batelaan, Anton J. L. M. van Balkom, Digna J. F. van Schaik, Patricia van Oppen, Brenda W. J. H. Penninx

**Affiliations:** 1 Amsterdam UMC, Vrije Universiteit, Psychiatry, Amsterdam Public Health Research Institute, Amsterdam, The Netherlands; 2 GGZ inGeest Specialized Mental Health Care, Amsterdam, The Netherlands

**Keywords:** Depression, Anxiety, Treatment, Antidepressant, SSRI, Running therapy, Aging, Telomere length, Telomerase activity, Inflammation, Metabolic syndrome, Cortisol, fMRI

## Abstract

**Background:**

Depressive and anxiety disorders have shown to be associated to premature or advanced biological aging and consequently to adversely impact somatic health. Treatments with antidepressant medication or running therapy are both found to be effective for many but not all patients with mood and anxiety disorders. These interventions may, however, work through different pathophysiological mechanisms and could differ in their impact on biological aging and somatic health. This study protocol describes the design of an unique intervention study that examines whether both treatments are similarly effective in reducing or reversing biological aging (primary outcome), psychiatric status, metabolic stress and neurobiological indicators (secondary outcomes).

**Methods:**

The MOod Treatment with Antidepressants or Running (MOTAR) study will recruit a total of 160 patients with a current major depressive and/or anxiety disorder in a mental health care setting. Patients will receive a 16-week treatment with either antidepressant medication or running therapy (3 times/week). Patients will undergo the treatment of their preference and a subsample will be randomized (1:1) to overcome preference bias. An additional no-disease-no-treatment group of 60 healthy controls without lifetime psychopathology, will be included as comparison group for primary and secondary outcomes at baseline. Assessments are done at week 0 for patients and controls, and at week 16 and week 52 for patients only, including written questionnaires, a psychiatric and medical examination, blood, urine and saliva collection and a cycle ergometer test, to gather information about biological aging (telomere length and telomerase activity), mental health (depression and anxiety disorder characteristics), general fitness, metabolic stress-related biomarkers (inflammation, metabolic syndrome, cortisol) and genetic determinants. In addition, neurobiological alterations in brain processes will be assessed using structural and functional Magnetic Resonance Imaging (MRI) in a subsample of at least 25 patients per treatment arm and in all controls.

**Discussion:**

This intervention study aims to provide a better understanding of the impact of antidepressant medication and running therapy on biological aging, metabolic stress and neurobiological indicators in patients with depressive and anxiety disorders in order to guide a more personalized medicine treatment.

**Trial registration:**

Trialregister.nl Number of identification: NTR3460, May 2012.

## Background

Depressive and anxiety disorders are common comorbid conditions with a large impact on public health [1, 2]. Meta-analyses show that persons with depressive and anxiety disorders have increased risks for the onset of cardiovascular diseases, diabetes, stroke, obesity [3], and advanced physical [3, 4] and cognitive decline [5]. In other words, depressive and anxiety disorders need to be considered as an important risk factor for a multitude of aging-related conditions. Dysregulation of physiological stress systems such as inflammation, hyperactivity of the HPA-axis and metabolic dysregulation [6, 7] have been suggested to partly underlie these associations. Additionally, persons with depressive and anxiety disorders are found to be subject to advanced biological aging. Furthermore, these physiological stress systems may also play a role in recovery mechanisms of depression and anxiety disorders.

Two treatment regimens for depressive and anxiety disorders that have shown to be effective are antidepressants and running therapy [8–11]. It is, however, unclear whether they could beneficially affect biological aging and physiological stress-systems dysregulations. Well-designed studies looking into the underlying physiological pathways of both treatments are lacking. It has been suggested that these interventions may work through different pathophysiological mechanisms. Despite their comparable effectiveness on mental health outcomes [8], running therapy may have a more beneficial impact on somatic health indicators including biological aging [12]. This intervention study examines and compares the impact of antidepressant medication and running therapy on biological aging, metabolic stress and neurobiological abnormalities related to depression and anxiety.

### Depressive and anxiety disorders and biological aging

In line with their negative impact on a multitude of aging-related somatic conditions, depression and anxiety disorders have found to be related to more advanced biological aging. This is for instance evidenced by shorter telomeres found in depression and/or anxious patients as compared to healthy controls [13–16]. Telomere length (TL), a relatively well-studied marker of cellular age, integrates the cumulative lifetime burden of genetic and environmental factors dependent on chronological age [17], and predicts several aging-related diseases and early mortality [18]. Telomeres are tandem repeated DNA sequences that form protective caps at chromosome ends [19] which can be elongated by telomerase enzymes. High telomerase activity has protective functions for aging and cell death and lower telomerase activity is linked to aging-related disease factors [20–22]. Some studies suggest that telomerase activity is elevated in the presence of a depression diagnosis [23], possibly as an attempt to compensate for the loss of TL. However, another study found decreased telomerase activity in a chronically stressed sample [24], leaving it unclear whether increased activity of the enzyme is a sign of improved health or rather a compensatory mechanism. The extent to which depression and anxiety treatment impacts the telomere/ telomerase system has not been extensively examined [25].

### Depressive and anxiety disorders, metabolic stress and neurobiological abnormalities

In various studies and meta-analyses, depressive and anxiety disorders have been linked to physiological alterations of central bodily stress systems: systemic inflammation [7, 26, 27] and oxidative stress [6], hyperactivity of the hypothalamus-pituitary adrenal (HPA) axis [28], a dysregulated autonomic tone [29, 30] accompanied with metabolic syndrome dysregulations [31]. Metabolic and physiological stress system dysregulations could contribute to the process of advanced biological aging as they have shown to affect TL and the telomere maintenance system [32–34].

Physiological stress systems also impact the structural and functional integrity of the brain, such as hippocampal volume, prefrontal cortex (PFC) morphology, and activity of the amygdala, insula and anterior cingulate cortex (ACC) [35–38], albeit inconsistently [39]. These are key brain regions implicated in depression and anxiety as there is converging evidence for widespread but subtle structural alterations in prefrontal regions such as the ACC, dorsomedial and orbitofrontal cortex, posterior cingulate cortex, insula, and the hippocampus [40–45]. There is also some evidence for rostral ACC, amygdala and medial PFC hyperactivation during emotional processing, while dorsal regions may be hypoactive in people with depression or anxiety disorders [46–49], although findings have been inconsistent across studies [50].

### Depressive and anxiety disorder treatment and physiological changes

Commonly prescribed selective serotonin re-uptake inhibitors (SSRI) have shown to be effective in depression and anxiety treatment [9, 51]. Some -although limited- evidence exists suggesting that SSRI treatment results in decreased cortisol [52], inflammatory [53] and antioxidant [54] levels. A recent review suggested a role for telomerase activity mediating the beneficial effects of antidepressants medication [55], possibly by promoting cell survival and/or function both in the brain and in the periphery. Only a few studies examined the association between antidepressant treatment and the telomere system and found shorter leukocyte telomere length (LTL) in patients who did not respond to antidepressants compared to those who did respond [56, 57]. Sample sizes of above-mentioned studies were relatively small, thus associations between antidepressant response and telomere length/telomerase activity remain to be extensively explored.

A similarly effective intervention is running therapy [10, 58, 59]. Running therapy works through the direct impact of aerobic exercise on opioid [60, 61], monoaminergic mechanisms [62] and regional cerebral blood flow [63]. The impact of running therapy has also been shown to reduce oxidative stress [64], inflammation [65–67], and cortisol [68]. Exercise has also shown to have beneficial impact on TL in a cancer population with higher telomerase activity emerging after 3 months of exercise, which was paralleled by decreases in psychological distress [69], a finding confirmed in other research [12, 70–72]. Two studies comparing running therapy and SSRI treatment confirmed a similar effectiveness for depression [8, 73] and anxiety disorders [74]. Nevertheless, these interventions probably work through different pathophysiological mechanisms and may have different impact on biological aging.

### Objective

This intervention study examines and compares the impact of antidepressant medication and running therapy on biological aging (primary outcome) and psychiatric status, metabolic stress and neurobiological abnormalities relevant for depression and anxiety disorders (secondary outcomes). This study also examines to what extent treatment-induced improvement in psychiatric status parallels with improvement of biological aging, metabolic stress and neurobiological abnormalities. Furthermore, this study compares the pre- and posttreatment outcomes to the physiological stress parameters of the no-disease-no-treatment control group.

## Methods

### Study design

**Fig. 1:**
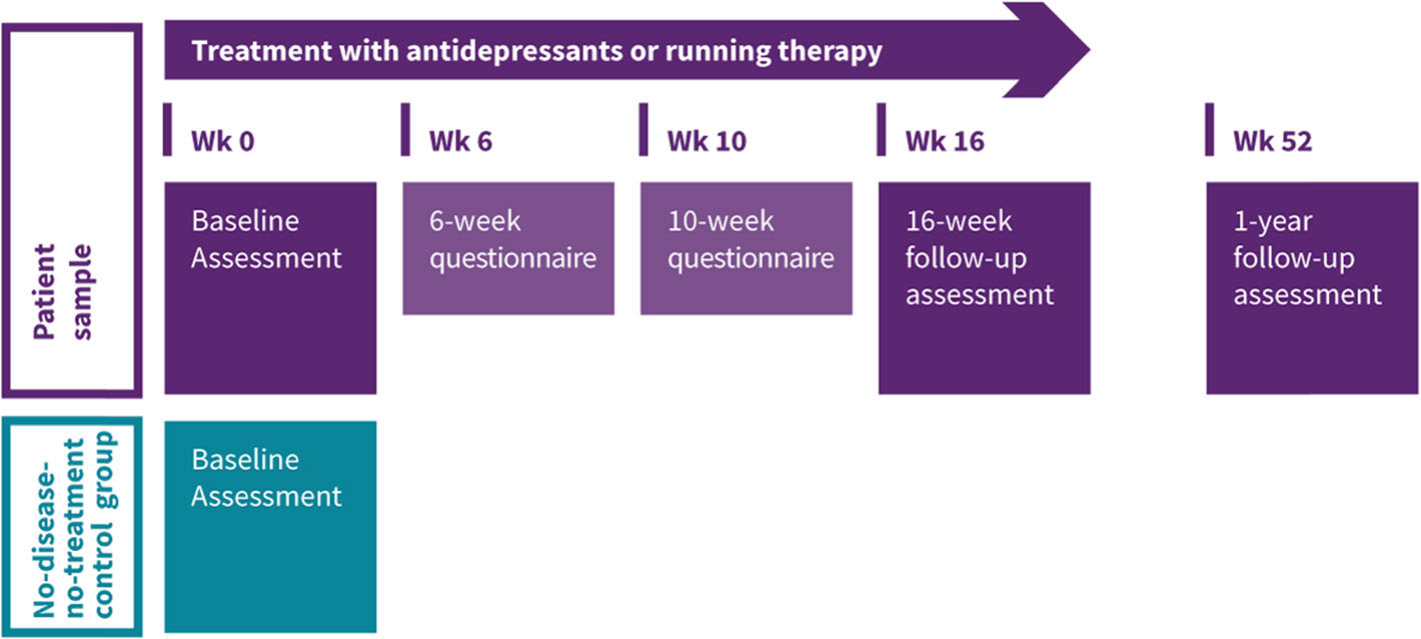
MOTAR flowchart

The MOod Treatment with Antidepressant or Running (MOTAR) study is a 16-week intervention study with two treatment arms: 1) antidepressant medication and 2) running therapy (see Fig. 1). In total, 160 patients with a depressive and/or anxiety disorder receive antidepressants or running therapy. Depressive and anxiety disorders are highly comorbid [75], also over time [76, 77], their underlying pathophysiology is largely comparable and both disorders are treated with similar treatments [10, 51, 74, 78]. A randomised controlled trial is the preferred method to compare two interventions, but also comes with limitations: quite some patients do not agree with random treatment assignment, and therefore, studies may result in selective inclusion of subjects which hampers the generalizability of results. Consequently, we decided to conduct a pragmatic study (resembling a partially randomised preference patients design (PRPP) [79]. First, patients without strong preference for treatment allocation are randomly allocated (1:1) to either antidepressant medication or running therapy. The SPSS random generator (SPSS, version 20.0) is used to randomise these participants. Subsequently, persons who were not willing to be randomised but are willing to participate in the study, were allocated to their preferred intervention. In order to be certain that no age differences arise, randomization is stratified by age in two groups (cut off 40 years). Further, in a subset of at least 50 subjects (25 from both treatment conditions) neuroimaging (Magnetic Resonance Imaging (MRI) data will be collected. A no-disease-no-treatment-control group (*N* = 60) will be examined to compare health, physiological and neurobiological indicators between persons with and without depression and anxiety disorders at baseline, and allows checks on whether improvements over time after treatment completely restores health and physiological levels to those of healthy controls.

### Recruitment and study settings

Between 2012 and 2019, patients are recruited when (newly) enrolled at GGZ inGeest (mental health organization in the surroundings of Amsterdam, The Netherlands) with depressive and/or anxiety disorders. Patients receive information about the study during the intake and are asked for their participation. During a telephone screening, in- and exclusion criteria are checked and when consent is given, patients undergo a baseline assessment before starting their treatment. All patients are also asked to participate in the MRI study substudy.

The no-disease-no-treatment-control group is recruited through advertisements in the area and through the website www.motar.nl. Persons receive information about the study and are asked for their participation. Patients and controls are matched on the basis of age, sex and educational level. In- and exclusion criteria are checked and after given consent, the healthy persons only undergo a baseline and neuroimaging assessment.

### Eligibility criteria

Inclusion criteria of the patient sample include: having a current depressive disorder (major depressive disorder) or anxiety disorder (social phobia, generalized anxiety disorder, panic disorder with or without agoraphobia) as ascertained by the Diagnostic and Statistical Manual of Mental Disorders – Fourth edition (DSM-IV) algorithms with the CIDI (Composite International Diagnostic Interview) [80] and being aged between 18 and 70 years. Exclusion criteria are: 1) use of antidepressants in last 2 weeks, 2) current use of other psychotropic medication, except for the use of benzodiazepines with stable usage, 3) regular exercising more than once a week, 4) primary severe, clinically diagnosed psychiatric diagnosis other than a depressive or anxiety disorder, 5) evidence of acute suicidal risk (based on clinical view), 6) medical contra-indications to running therapy or antidepressants (e.g. serious heart problems) as confirmed by the patient’s physician, and 7) being pregnant.

Inclusion criteria of the no-disease-no-treatment-control group are having a negative lifetime history of psychiatric disorders as checked with the CIDI and being aged between 18 and 70 years. Exclusion criteria are: 1) participation in regular (> 1/week) exercise and 2) medical contra-indications to running therapy or antidepressants (e.g. serious heart problems) as confirmed by a physician.

Additional exclusion criteria for the MRI sub-study are major internal or neurological disorders, pregnancy and known contra-indications for MRI investigations, such as the presence of metal objects (e.g. pacemaker, arteriovenous clips) or claustrophobia.

### Consent procedure, baseline and follow-up assessments

Informed consent approved by the Medical Ethical Committee VU University Medical Centre has to be signed before starting the baseline assessment. During a 4-h face-to-face baseline assessment a wide range of data will be collected, including demographic information, a diagnostic psychiatric interview, a medical examination, a cycle ergometer test, collection of saliva, urine, and blood and various self-reported clinical questionnaires. At week 6 and week 10 depression and anxiety symptom severity will be assessed by self-report questionnaires. At week 16 and week 52, the assessments will be repeated in the patient sample (see Fig. 1). For each face to face assessment, the patient will receive a gift voucher of €50. MRI measurements consist of a clinical interview and a neuroimaging session with a total duration of approximately 2.5 h. For each MRI measurement, the patient will receive a gift voucher of €25-. The control group will undergo a baseline and neuroimaging assessment, but no follow-up assessments. The control participants will receive a gift voucher of €50. Table 1 gives an overview of the data collection.

**Table 1:** Collected information on central (mental) health outcomes in MOTAR

	Instrument	Method	Week 0	Week 6	Week 10	Week 16	Week 52
Primary outcomes							
Biological aging: telomere length, telomerase activity	Fasting blood samples	Blood	X	–	–	X	X
Secondary outcomes							
Biological and general health indicators							
Biomarkers (inflammation, metabolic syndrome)	Fasting blood samples	Blood	X	–	–	X	X
Gene-expression (RNA)	Fasting blood samples	Blood	X	–	–	X	X
HPA-axis (cortisol)	2 days of 6 saliva samples	Saliva	X	–	–	X	X
Oxidative stress	Urine sample	Urine	X	–	–	X	X
Autonomic nervous system	Electro + impedance cardiography, heart rate variability [81]	ME	X	–	–	X	X
Blood pressure	Systolic and diastolic BP	ME	X	–	–	X	X
Body composition	Weight, height, waist+ hip circumfereence	ME	X	–	–	X	X
Physical condition	Astrand sub max test [82]	ME	X		–	X	X
Muscle strength	Hand grip strength [81]	ME	X	–	–	X	X
Lung function	Peak expiratory flow [82]	ME	X	–	–	X	X
Pain	Chronic graded pain scale [83]	Int	X	–	–	X	X
Somatization	Short somatization questionnaire [84]	SR	X	–	–	X	X
Disability severity	WHO-DAS II [85]	SR	X	–	–	X	X
Depressive and anxiety disorders							
Presence of MDD	CIDI: MDD [78]	Int	X	–	–	X	X
Presence of anxiety dis	CIDI: SocPhob, Agora, GAD, PA [78]	Int	X	–	–	X	X
Course of symptoms	Life-chart [86]	Int	X	–	–	X	X
Severity of depression	Inventory of depressive symptoms [87]	SR	X	X	X	X	X
Severity of anxiety	Beck anxiety index [88] and Fear questionnaire [89]	SR	X	X	X	X	X
Sleep	Insomnia Rating Scale [90]	Int	X	–	–	X	X
Descriptive variables, potential confounding covariates and potential mediating variables							
Age, gender, ethnicity	Standard questions	Int	X	–	–	–	–
Socio-economic status	Education, income, occupation	Int	X	–	–	–	–
Physical activity	SQUASH questionnaire [91]	SR	X	–	–	X	X
Smoking	Past + current smoking questions	SR	X	–	–	–	X
Medication use	Drug container observation	Int	X	–	–	X	X
Regular alcohol intake	AUDIT questionnaire [92]	SR	X	–	–	–	X
Somatic diseases	Presence + symptoms of disease	Int	X	–	–	X	X
Health care	Perceived need of care [93]	Int	X	–	–	X	X
Work and disability	Tic-P questionnaire [94]	Int	X	–	–	X	X
Personality	NEO-FFI questionnaire [95]	SR	X	–	–	X	X
Locus of control	Pearlin & Schooler mastery scale [96]	SR	X	–	–	X	X
Depression vulnerability	LEIDS-R questionnaire [97]	SR	X	–	–	X	X
Anxiety vulnerability	Anxiety senstivity index [98]	SR	X	–	–	X	X
Experimental cognitive task	Implicit association test (IAT) [99]	CT	X	–		X	X
Experimental memory task	Digit Span (WAIS) [100]	Int	X	–	–	X	X
Important neg + pos life events	Brugha questionnaire [101]	Int	X	–	–	X	X
Childhood Trauma	Youth Trauma questionnaire [102]	SR	X	–	–	–	–
Familiy history	Familiy history inventory	Int	X	–	–	–	–
Neuroimaging assessment (subsample)							
Verbal episodic memory	15-words test [103]	MRI	x	–	–	x	x
Task-related brain activity	Emotional face matching paradigm and N-back paradigm [104]	MRI	x	–	–	x	x
Brain network connectivity	Resting state MRI images	MRI	x	–	–	x	x
Process indicators (intervention adherence)							
Exercise intervention group: Exercise participation and heart rate will be administrated during each session..							
Antidepressant intervention group	Side effect medication questionnaire	Int	–	X	X	X	–
	Adherence (pill count)	Int	–	X	X	X	–

*SR* self-report, *Int* interview, *Blood* data collection via fasting blood sample, *CT* computer task, *ME* medical examination

### Intervention

Participants will undergo an intervention of 16 weeks since this period has shown to be sufficient to decrease depressive and anxiety symptoms and to impact on physiological stress after antidepressant therapy [101] or running therapy [102, 103].

#### Antidepressant medication

Patients will receive standardized treatment with escitalopram, a selective serotonin reuptake inhibitor (SSRI) which has documented efficacy, a rather favorable side effect profile, is recommended as first-step treatment in both the General Practitioner (NHG Standardized depressive disorder and anxiety disorder (in Dutch)) and Psychiatry treatment guidelines (Multidisciplinary guidelines depression and anxiety (in Dutch)), and is one of the most commonly prescribed antidepressants [101, 104]. An initial dosage of 10 mg per day of escitalopram is used. Medication management is provided by a psychiatrist who meets each patient at study onset and at weeks 2, 6, 10 and 16. At these meetings, the psychiatrist evaluates treatment response and side effects, and titrates dosage (to a maximum of 20 mg) according to the multidisciplinary depression/anxiety guidelines until a clinically effective dosage is achieved. Following the medication protocol, if the initial SSRI is poorly tolerated, the psychiatrist can switch prescription to another SSRI drug (sertraline, dosage of 50 mg to a maximum of 150 mg). Adherence to treatment is evaluated by a patient’s diary and administration log by the psychiatrist. After 16 weeks of treatment, a research assessment will take place and further treatment is conducted following clinical guidelines.

#### Running therapy

Therapy consists of three 45-min outdoor running sessions per week, in line with the public health recommendations by CDC/American College of Sports Medicine [105] and its earlier successful effects on depression and anxiety [74, 106]. Patients will be gradually assigned individual training ranges equivalent to 70–85% of their heart rate reserve, calculated from the heart rate achieved during a cycle ergometer test with the formula of Karvonen [107]. This intensity level was confirmed to be effective in decreasing depressive symptoms [103]. During the screening phase and during baseline assessment, so before formal inclusion to the study, potential physical and/or somatic problems and use of medication are administrated. When serious somatic conditions are signalled, the person’s own physician will be contacted and consulted in order to discuss potential study participation. Furthermore, at the beginning of the running intervention, the running therapist discusses experience of exercise in the past, and will provide information about food, moisture balance, fatigue, injuries, sleep and recovery. The running therapy intervention was conducted at a medical institution (GGZinGeest) where there is always a physician approachable.. Running sessions will be organized and supervised by qualified staff, starting with a 10-min warming-up exercise period followed by 30 min of jogging at an intensity that maintains heart rate within the assigned training range (starting in the first 4 weeks at 50–70% of heart rate reserve and in the subsequent 12 weeks at 70–85% of heart rate reserve), finishing with 5 min of cooling-down exercises. During the running sessions, all subjects wear a heart rate monitor. Heart rate will be confirmed three times per session to ensure that patients are exercising within the prescribed exercise training ranges. Data of the heart rate monitor will be uploaded after sessions and used to encourage study compliance. Patients are stimulated to participate in all three organized group sessions, but if strongly preferred, home-based individual running is allowed once per week. The trainer monitors training attendance. The size of the running group is on average 5 or 6 patients. Both interventions were conducted using evidence-based clinical guidelines (https://www.nhg.org/sites/default/files/content/nhg_org/uploads/multidisciplinaire_richtlijn_depressie_3e_revisie_2013.pdf). Adverse events in both treatment programs will be signalled and reported the medical ethical committee. After 16 weeks of treatment, a research assessment will take place and further treatment is conducted following clinical insights by the responsible clinician.

### Outcomes

#### Primary outcomes

The primary outcome of this trial is the change in biological aging, measured through TL and telomerase activity before the start and at the end of the intervention. TL has been shown to be correlated to functioning of multiple physiological stress systems such as the immune-inflammatory system, the hypothalamus-pituitary-adrenal (HPA)-axis and the autonomic nervous system (ANS) [33, 34] and therefore picks up potential improvement in various underlying mechanisms. In addition, TL has been shown to be predictive of various somatic health outcomes including mortality. TL has earlier been used in studies examining the effects of lifestyle interventions [72, 108, 109] and has shown sensitive to change, even at rather short term, interventions were linked to less shortening of TL. In addition to explore the underlying telomere system dynamics, we also will measure telomerase activity, as was done in Wolkowitz et al. (2012) [23]. TL will be measured from purified DNA samples from peripheral blood mononuclear cells that were stored frozen at − 80 °C using a quantitative polymerase chain reaction (qPCR)-based assay. Telomerase enzymatic activity will be measured by the Telomerase Repeat Amplification Protocol (TRAP149) using the commercial TRAPeze kit (Chemicon, Upstate/CHEMICON, Temecula, CA, USA) [23]. Less shortening of TL after treatment will be seen as reverse of biological aging.

#### Secondary outcomes

##### Biological and general health indicators

Biomarkers of physiological health will be gathered through fasting blood samples, 24-h urine, and six saliva samples were taken at 1 day covering morning awakening response (at awakening and at 30, 45 and 60 min later), afternoon (at 6 pm) and evening levels (at 10 pm) to e.g. examine inflammatory markers, cortisol levels, metabolic syndrome abnormalities, DNA and oxidative stress. Activity of the autonomic nervous system will be measured using the ambulatory monitoring system (VU-ams) of which reliability and recording methodology have been described previously [110]. Furthermore, blood pressure, fitness (using bicycle ergometer with the Astrand method [111]), hand grip strength (by Jamar hand grip meter) [112] and lung function (using Mini Wright peak flow meter) [113] will be tested. The chronic graded pain scale [114] will be taken to evaluate pain, somatization will be assessed with the short somatization scale [115] and disability severity will be gathered using the WHODAS II [116].

##### Depressive and anxiety disorders

The presence of depressive disorders (Major Depressive Disorder) and anxiety disorders (social phobia, generalized anxiety disorder, panic disorder and agoraphobia) will be established using the CIDI. The CIDI is a valid and reliable instrument to assess depressive and anxiety disorders [80] and will be administered by specially trained research staff. The type and number of depressive and anxiety disorders will be compared across the intervention groups and, if necessary, these clinical characteristics will be considered as covariates in the main analyses. Fluctuation of depression and anxiety during follow-up will be examined using the Lifechart method [81]. Severity of depression is measured using the 30-item Inventory of Depressive Symptomatology (IDS-SR30) [82]. Severity of anxiety is measured with the 21-item Beck Anxiety Inventory (BAI) [83]. For both scales higher scores mean higher symptom severity. Phobia symptoms will be measured with the Fear Questionnaire [84]. Sleep duration and quality will be examined with the Insomnia Rating Scale [85]. Psychotropic medication use was assessed during the interview at baseline, 16 and 52 weeks by inspection of the participant’s medication containers. It contained lifetime history of use as well as use during the study and was classified using the World Health Organization Anatomic Therapeutic Chemical (ATC) classification (World Health Organization Centre for Drug Statistics Methodology, 2010).

##### Descriptive variables, potential confounding covariates and potential mediating variables

Lifestyle indicators, (change in) health care status and utilization, personality and cognitive vulnerability, and personal history are also considered as mediating variables. The SQUASH questionnaire [86] will be taken to examine daily-life physical activities. Questions of smoking and drug use will be asked during the interview and the Audit questionnaire [87] will be used to measure regular alcohol intake. A chronic disease inventory and the Perceived Need for Care Questionnaire will be used to assess (changes in) health and health care use [88]. Loss of productivity at work and health care utilization will be gathered with the TIC-P [89]. Personality and cognitive vulnerability traits will be measured using the NEO-FFI questionnaire [90], personal mastery questionnaire [91], the Leids-R questionnaire [92], and the Anxiety Sensitivity Index (ASI) [93]. The Implicit association test (IAT) [94] will be used as experimental cognitive emotional task and the Digit Span (WAIS) [95] as an experimental memory task to assess working memory. Personal history contains assessment of important negative life events with the Brugha questionnaire [96], childhood trauma with the childhood trauma questionnaire [97], and family history of psychiatric disorders will be gathered by specific questions.

##### Neuroimaging assessment

In a subsample of the patients and in the healthy controls a neuroimaging assessment will be taken using the 3 T Philips Intera MR system*.* The 15-words test, a Dutch version of the Rey’s auditory verbal learning test [98] will be performed outside the scanner to assess verbal episodic memory.

Anatomical T1-weighted and diffusion tensor imaging (DTI) scans will be obtained to assess grey and white matter structure. An emotional face matching paradigm [99] and N-back paradigm [100] will be employed to examine task-related brain activity. Finally, brain network connectivity will be examined during rest by acquiring resting state fMRI images.

### Sample size

Published running therapy and antidepressant intervention studies in non-psychiatric groups have yielded effect sizes for changes in biological aging ranging from 0.5 [74] to 1.2 [112]. When using the minimum effect size found (0.5), 80% power and *p* = 0.05, we need 63 subjects per group. Considering a dropout of 20%, *n* = 76 patients per group are needed to illustrate significant antidepressant and running therapy effects on biological aging in a patient group. That is why we strive for 80 patients per group, and 160 total.

As described by Thirion, functional MRI analyses require a minimum of 25 subjects per group for adequate statistical power [117]. In addition, we aimed to include 60 healthy controls to allow additional comparisons in outcomes between controls and patients.

### Organisation, and quality insurance and data management

Compliance with antidepressant medication or running therapy is assessed using patient’s and therapeutic logs. Patients who withdraw from the intervention will be asked reason(s) for drop out and they will be motivated to continue the measurements with the purpose to minimize loss of follow-up data and to make the intention to treat analysis and per-protocol analysis possible.

Research data will be collected by a coded participant number. Interviews will be conducted by computer and questionnaires by paper and will be entered into the system by the research assistant. An administrative database will be used to ensure timely assessments. The data manager will make back-ups for the monitoring of overall progress and data quality.

### Statistical analysis plan

Missing data will be inspected and handled via full information maximum likelihood. Mixed model regression analyses will be conducted to estimate the effect size of both.

Interventions on biological aging and psychiatric status, metabolic stress and neurobiological abnormalities. Per protocol analyses within intervention groups will be conducted to evaluate whether change of biological aging and metabolic stress is a function of protocol adherence. The two intervention groups will be compared using mixed models or generalized estimating equations (GEE) to assess the longitudinal change of biological aging, physiological and metabolic stress and psychiatric symptoms. These models will also compare physiological and clinical effects of those who are willing and not willing to be randomised to check the impact of a patients’ preferred or allocated intervention. Furthermore, pre- and posttreatment outcomes to physiological stress parameters will be compared to the no-disease-no-treatment control group using regression analyses.

### Trial status

The MOTAR study was approved by the Medical Ethics Committee of the VU University Medical Center Amsterdam and registered with the Netherlands Trial Register under NTR3460. Recruitment commenced in September 2012 and is ongoing.

## Discussion

A wide range of treatment programs for depressive and anxiety disorders are available but it remains largely unknown whether the impact of these programs on biological aging, metabolic stress and neurobiological abnormalities are comparable. Treatment with antidepressant medication or running therapy have both shown to be effective in depression and anxiety, but a well-designed comparative study of these treatment strategies and their impact on physiological and neurobiological processes is currently lacking. As the number and type of clinician contacts between groups are not similar, this could be underlying clinical improvement. However, the interventions in this trial were developed in line with current guideline standards and therefore are as much as possible reflective of regular clinical care treatments.

This intervention study is designed to examine and compare the impact of antidepressant medication and running therapy on changes of both mental and physiological health, including biological aging, metabolic stress and neurobiological function and whether these pre- and posttreatment outcomes are comparable with persons without a psychiatric status. It is expected that this study provides more detailed information about underlying biological mechanisms of depression and anxiety treatment effects. Having insight in the favourable physiological stress effects of these treatment regimens could probably also be helpful in increasing the effectiveness of personalised medicine.

## Data Availability

MOTAR-data can be requested through the submission of an analysis plan. Instructions can be found on the website www.motar.nl

## Abbreviations

ACC: Anterior cingulate cortex
Agora: Agoraphobia
ASI: Anxiety sensitivity index
BAI: Beck’s anxiety index
BP: Blood pressure
CDC: Center for disease control
CIDI: Composite international diagnostic interview
CT: Computer task
DSM-IV: Diagnostic and statistical manual of mental disorders fourth edition
DTI: Diffusion tensor imaging
GAD: Generalised anxiety disorder
HPA: Hypothalamus-pituitary adrenal
IAT: Implicit association task
IDS-SR: Inventory of depressive symptomatology
LTL: Leukocyte telomere length
MDD: Major depressive disorder
ME: Medical examination
MOTAR: Mood treatment with antidepressants or running
MRI: Magnetic resonance imaging
NEO-FFI: Neuroticism- extraversion-openness five-factor inventory
NHG: Nederlands huisartsen genootschap
PA: Panic disorder
PFC: Prefrontal cortex
qPCR: Quantitative polymerase chain reaction
SocPhob: Social phobia
SPSS: Statistical package for social sciences
SQUASH: Short questionnaire to assess health enhancing physical activity
SR: Self-report
SSRI: Selective serotonin re-uptake inhibiters
TIC-P: Treatment inventory of costs in patients
TL: Telomere length
TRAP: Telomerase repeat amplification protocol
VU-ams: VU university ambulatory monitoring system
WAIS: Wechsler adult intelligence scale
WHODAS: World health organisation disability assessment schedule
